# The impact of the COVID-19 pandemic on a cohort of Labrador retrievers in England

**DOI:** 10.1186/s12917-022-03319-z

**Published:** 2022-06-24

**Authors:** Charlotte S. C. Woolley, Ian G. Handel, B. Mark Bronsvoort, Jeffrey J. Schoenebeck, Dylan N. Clements

**Affiliations:** The Roslin Institute and Royal (Dick) School of Veterinary Studies, The University of Edinburgh, Easter Bush Campus, Midlothian, EH25 9RG United States

**Keywords:** Epidemiology, Labrador retrievers, Animals, Dogs, COVID-19, Pandemics, Cohort studies, Longitudinal studies, Veterinarians, Surveys and questionnaires

## Abstract

**Background:**

The COVID-19 pandemic is likely to have affected the welfare and health of dogs due to surges in adoptions and purchases, changes in the physical and mental health and financial status of dog owners, changes in dogs’ lifestyle and routines and limited access to veterinary care. The aims of this study were to investigate whether COVID-19 restrictions were associated with differences in Labrador retrievers’ lifestyle, routine care, insurance status, illness incidence or veterinary attendance with an illness, who were living in England and enrolled in Dogslife, an owner-based cohort study. Longitudinal questionnaire data from Dogslife that was relevant to the dates between the 23rd of March and the 4th of July 2020, during COVID-19 restrictions in England, were compared to data between the same dates in previous years from 2011 to 2019 using mixed regression models and adjusted chi-squared tests.

**Results:**

Compared with previous years (March 23rd to July 4th, 2010 to 2019), the COVID-19 restrictions study period (March 23rd to July 4th 2020) was associated with owners reporting increases in their dogs’ exercise and worming and decreases in insurance, titbit-feeding and vaccination. Odds of owners reporting that their dogs had an episode of coughing (0.20, 95% CI: 0.04–0.92) and that they took their dogs to a veterinarian with an episode of any illness (0.58, 95% CI: 0.45–0.76) were lower during the COVID-19 restrictions compared to before. During the restrictions period, owners were less likely to report that they took their dogs to a veterinarian with certain other illnesses, compared to before this period.

**Conclusions:**

Dogslife provided a unique opportunity to study prospective questionnaire data from owners already enrolled on a longitudinal cohort study. This approach minimised bias associated with recalling events prior to the pandemic and allowed a wider population of dogs to be studied than is available from primary care data. Distinctive insights into owners’ decision making about their dogs’ healthcare were offered. There are clear implications of the COVID-19 pandemic and associated restrictions for the lifestyle, care and health of dogs.

**Supplementary Information:**

The online version contains supplementary material available at 10.1186/s12917-022-03319-z.

## Background

The emergence of a novel coronavirus (SARS-CoV-2) in December 2019 and the subsequent COVID-19 pandemic that swept across nations worldwide led governments in many countries to impose social restrictions to control the spread of the virus [1, 2]. In the UK, a national lockdown was implemented on the 23rd of March 2020, where people were not permitted to leave their homes without a reasonable excuse [3]. These strict rules were eased over subsequent months, but the long-term impact of the pandemic itself and these associated control measures are not yet fully understood. In 2021, the Pet Food Manufacturers’ Association (PFMA) estimated that there were 12.5 million dogs in the UK, with one third of households owning a dog [4]. Aspects of the COVID-19 pandemic that are likely to have affected the welfare of pet dogs include surges in pet adoptions and purchases [5–7], the physical and mental health of their owners, changes in their lifestyle and routine, the financial status of their owners and limited access to veterinary care [8, 9]. Pandemics are becoming increasingly frequent and severe [10], so it is vital that we understand how biosecurity measures such as government imposed lockdowns impact on the lifestyles and health of companion animals, so that their welfare can be protected if similar restrictions are imposed in the future.

People with COVID-19 may be bedridden for weeks [11, 12] and unable to give their pets the quality of care that they usually receive. When dog owners die, are hospitalised or are forced to isolate, dogs may need to adapt to stressful new environments, people and routines [13, 14]. Many owners did not have a provisional care plan for their pet during the initial months of the pandemic, which may have led to sub-optimal care [15, 16]. Several human mental health implications of the COVID-19 lockdown restrictions have been reported [17–19]. Pet ownership during the pandemic has mostly been associated with positive mental health outcomes [20] but some owners felt concerned or stressed about caring for their pet [21–23] or felt their relationship with their pet became strained [20, 23]. The stress levels, emotional and physiological states of dog owners affect the stress levels, cognitive performance, and quality of life of their dogs [24–28] and owners with poorer mental health after lockdown reported more changes in their pets’ behaviour and welfare, both negative and positive [29].

COVID-19 lockdown restrictions in the UK led to a reduction in social interactions and differences in physical activity [30–34] and many people were furloughed (granted temporary leave from their employment) or worked from home [35]. Several surveys have reported changes to dogs’ routines, including decreased time left alone, differences in walking patterns and training and fewer opportunities to socialise with other dogs [16, 36–41]. Due to the strong attachment bond of pet dogs and their owners [42], increased contact would initially seem like a positive change, especially if they are usually left alone for extended time periods [43]. However, increased exposure to physical contact and decreased time exercising outside and socialising may exasperate behavioural difficulties in adult dogs or increase the chance of behavioural problems in puppies [44]. Several surveys have reported negative changes in dogs’ behaviour during COVID-19 lockdown restrictions [16, 40, 45–48]. A recent study that surveyed dog owners, during and post COVID − 19 lockdown restrictions, reported that a decrease in the time spent alone by dogs increased their risk of separation related behaviour [49]. Additionally, an increase in the frequency of dog bites was reported by paediatric departments during the 2020 lockdown in Italy [50], Canada [51] and the UK [52].

Veterinary practices were exempt from closing during the first UK lockdown but non-essential care was postponed [44, 53]. The pandemic might also have caused financial difficulty for some pet owners, meaning they could not afford veterinary bills or pet insurance [44, 54]. Results from a RCVS survey showed that in April 2020, 95% of veterinary practices saw a reduction in weekly practice turnover of 25% or more, 66% saw a reduction of 50% or more and 24% saw a reduction of 75% or more [55]. The Small Animal Veterinary Surveillance Network (SAVSNET) reported reductions in vaccination consultations during the first 2020 lockdown period and subsequent peaks in vaccine-controlled diseases such as parvovirus and ﻿leptospirosis. Reductions in consultations for gastroenteric and respiratory clinical signs, pruritis, trauma and tumours were also reported [56–58]. This indicates that owners were not seeking help as often as before the pandemic for non-emergency issues such as vaccinations, which can usually be delayed without causing harm but also potentially emergency issues such as traumas, which may have impacted dogs’ welfare.

Research has highlighted the problem of the “symptom iceberg” in medical consultation data: the missing information about the wider population that does not exist in healthcare records [59]. During a recent vomiting outbreak in dogs in the UK, the vomiting incidence rates reported by Dogslife, an owner-based longitudinal cohort study, were over double the equivalent vomiting consultation rates reported by SAVSNET [60, 61]. This can be explained by Dogslife reports showing that only one third of vomiting episodes led to a veterinary consultation [62]. Unfortunately, surveillance systems that rely on primary care data are particularly likely to be affected by bias during lockdown restrictions, due to vast decreases in the number of owners taking their pets to the veterinarian [63]. Therefore, it is impossible to distinguish whether reduced numbers of veterinary consultations were indicative of decreased illness incidence due to some protective measures of lockdown restrictions, fewer owners taking their dogs to the veterinarian with illness symptoms, or a combination of these factors. Furthermore, most research into changes in dogs’ lifestyles and routines during COVID-19 restrictions has been based on cross-sectional prospective surveys, which do not necessarily accurately estimate their extent. To the authors’ knowledge, no studies have reported on routine care, such as worming and treatment with anti-parasitic therapeutics during the pandemic and further research is needed to understand the implications of lifestyle changes on dogs’ physical health. Longitudinal cohorts established prior to the pandemic, such as Dogslife [64], provide an opportunity to study both lifestyle factors and illness incidence within the wider population before and after the pandemic, and thus, provide a more accurate estimation of the extent and impact of changes that occurred.

The first aim of this study was to investigate whether COVID-19 restrictions were associated with differences in Dogslife Labrador retrievers’ lifestyle (including exercise, dietary factors, bathing and sleeping habits), routine care (including worming, anti-parasitic treatments for fleas and ticks and vaccination) or insurance status. The second aim was to investigate whether COVID-19 restrictions were associated with differences in Dogslife Labrador retrievers’ illness incidence or veterinary attendance with an illness.

## Results

### The association of COVID-19 restrictions with the lifestyle and routine care of Dogslife dogs

**Fig. 1:**
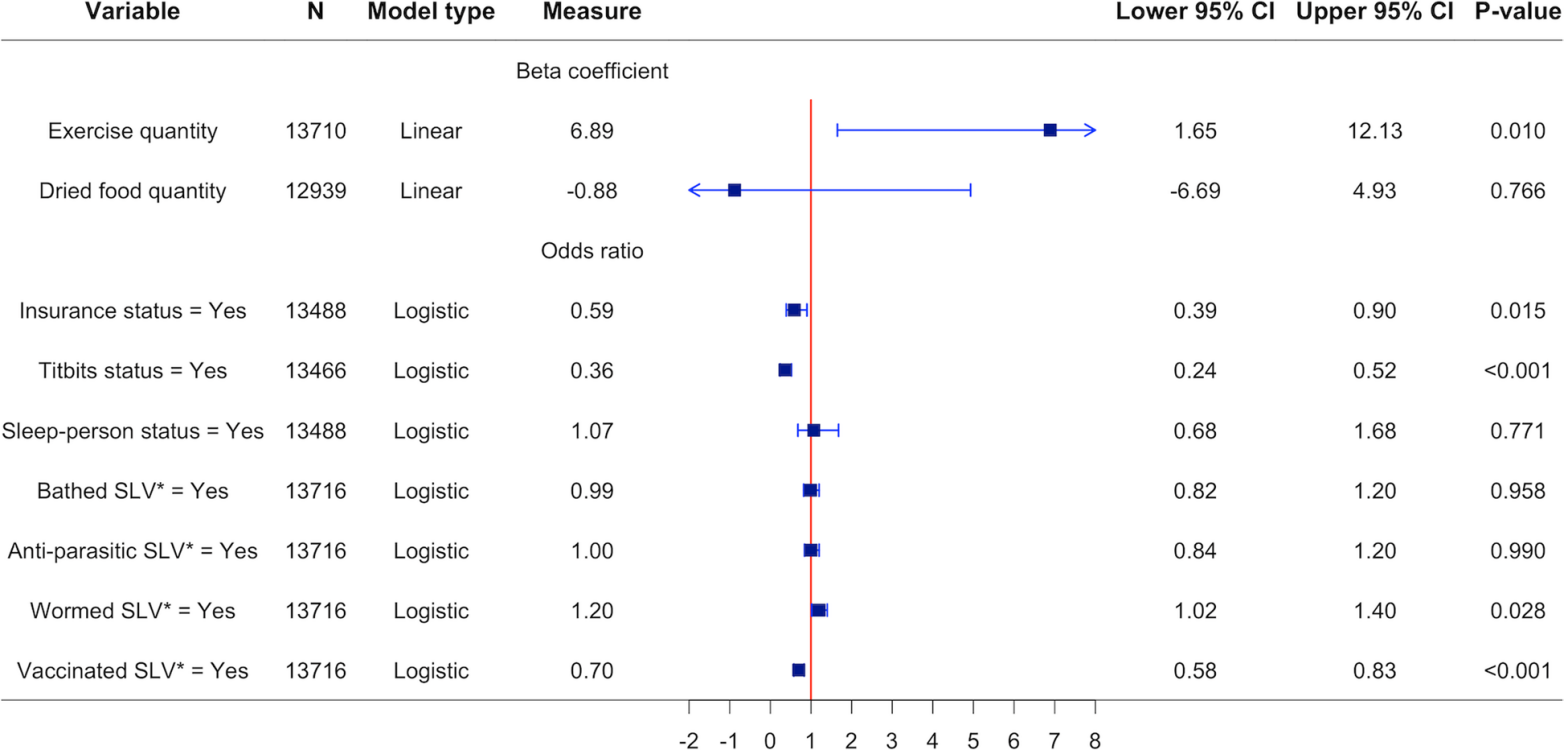
Forest plot of estimates from generalised additive mixed models. Contains the number of Dogslife questionnaires with data (N), the models’ type (linear or logistic), the models’ estimates for the beta coefficients (for linear models) or odds ratios (for logistic models) and associated 95% confidence intervals (CI) and *P*-values for comparing the COVID-19 restrictions study period (March 23rd to July 4th 2020) to data in the same date range in previous years (March 23rd to July 4th, 2010 to 2019) in each of the variables of interest. All models were adjusted for age, sex and individual effects. *Since the owner’s last visit to the Dogslife website (SLV)

To investigate the association of the COVID-19 restrictions with the lifestyle and routine care of Dogslife dogs, variables of interest from 13,716 questionnaires from 3889 dogs, entered into the Dogslife website by owners between March 23rd and July 4th, 2011 to 2020 were included in analysis. Summary statistics of the dogs in the questionnaires are given in Table 1. The data available from the questionnaires and the estimated beta coefficients and odds ratios for comparing the COVID-19 restrictions study period (March 23rd to July 4th 2020) to data in the same date range in previous years (March 23rd to July 4th, 2010 to 2019) from the linear and logistic generalised additive mixed effects models (GAMMs) respectively, adjusted for age, sex and individual effects for each the variables of interest are reported in the forest plot in Fig. 1.

**Table 1:** Summary statistics of Dogslife dogs (*N* = 3889)

Variable	COVID-19 restrictions study period (March 23rd to July 4th 2020)	Same date range in previous years (March 23rd to July 4th, 2010 to 2019)
Number of questionnaires	1265	12,451
Age category^a^		
Under 1 year 1 to 3.49 years 3.5 to 6.99 years 7 or over	124 (9.80%) 219 (17.31%) 418 (33.04%) 504 (39.84%)	5308 (42.63%) 3994 (32.08%) 2521 (20.25%) 628 (5.04%)
Sex		
Female Male	646 (51.07%) 619 (48.93%)	6134 (49.27%) 6317 (50.73%)
Working status		
Pet only Working/sporting/guide dog	1155 (91.38%) 109 (8.62%)	11,043 (90.52%) 1157 (9.48%)

^a^Dog age was calculated to the date that owners entered questionnaire data into the Dogslife website

The COVID-19 restrictions study period was associated with owners reporting an increase of 6.89 minutes per week (95% CI: 1.65–12.13) in the quantity of exercise dogs received; increased odds of reporting dogs being treated with a wormer since the owner’s last visit to the Dogslife website (SLV) (1.20, 95% CI: 1.02–1.40); decreased odds of reporting dogs being insured (0.59, 95% CI: 0.39–0.90), given titbits (0.36, 95% CI: 0.24–0.52) and being vaccinated SLV (0.70, 95% CI: 0.58–0.83). The COVID-19 restrictions study period was not associated with: owners reporting a difference in the quantity of dried food that dogs were fed, a change in odds of owners reporting their dogs sleep with a person at night, bathing dogs SLV or treating with an anti-parasitic SLV. An additional file 1 provides further details of the models’ selection, diagnostics, fit and full output.

### The association of COVID-19 restrictions with illness incidence and associated veterinary attendance in Dogslife

**Fig. 2:**
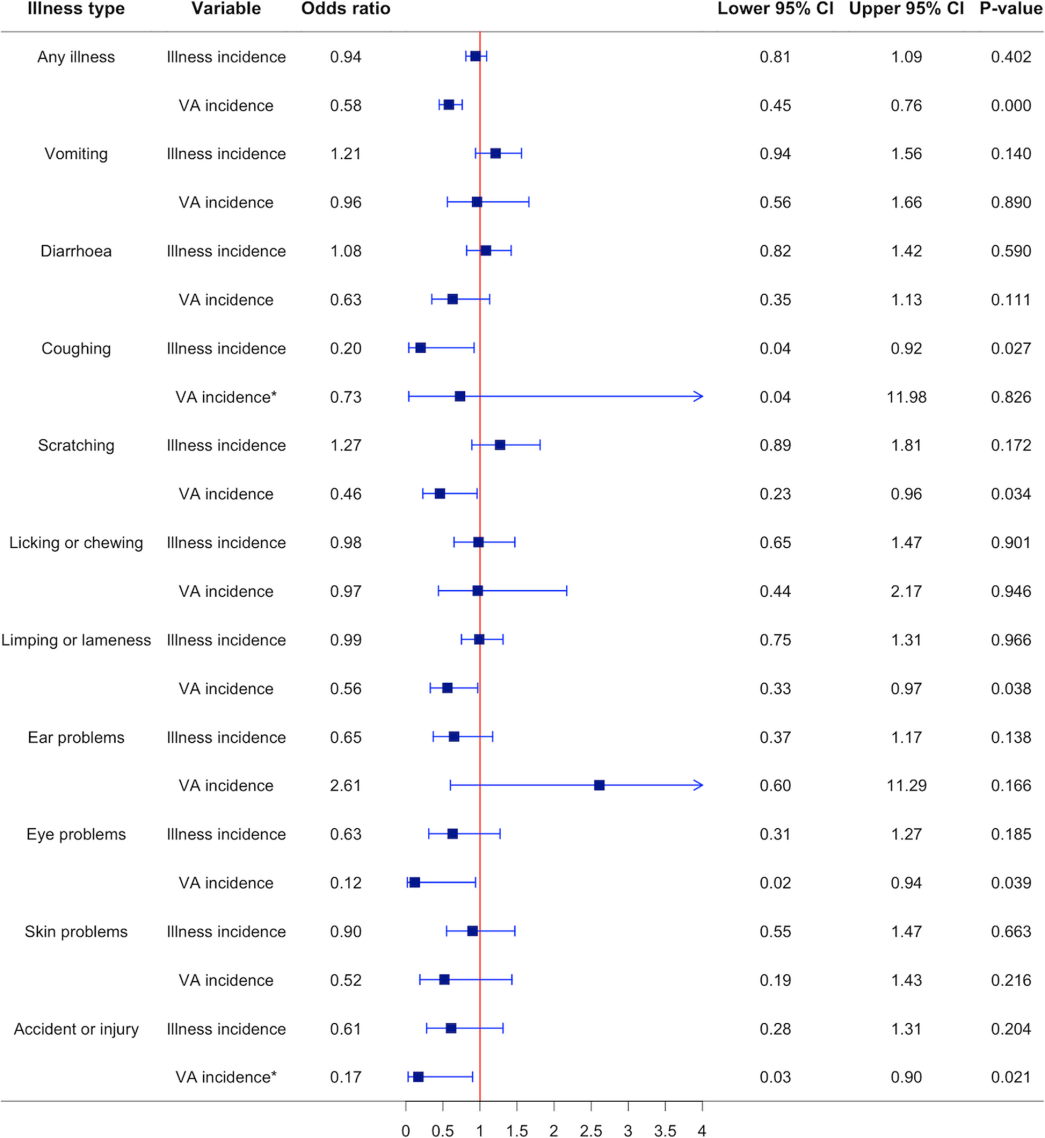
Forest plot of estimates from chi-squared tests. Contains the age-adjusted chi-squared tests’ estimates for the odds ratios and associated 95% confidence intervals (CI) and *P*-values for comparing the Dogslife illness incidences and veterinary attendance (VA) with illness incidences in the COVID-19 restrictions study period (March 23rd to July 4th 2020) to data in the same date range in previous years (March 23rd to July 4th, 2010 to 2019). *The Mantel-Haenszel adjustment for age was not performed on these variables due to expected frequencies of the stratified groups containing values less than 5

To investigate the association of the COVID-19 restrictions with Dogslife illness incidences and veterinary attendance with illness incidences, illness data from 16,115 questionnaires from 4110 dogs, entered into the Dogslife website by owners between March 23rd and July 23rd, 2011 to 2020 were included in analysis. Of these questionnaires, 3320 recorded at least one incidence of illness and 1850 recorded at least one incidence where owners had taken their dogs to the veterinarian with an illness. The estimated odds ratios for comparing Dogslife illness incidences and veterinary attendance with illness incidences in the COVID-19 restrictions study period to data in the same date range in previous years from chi-squared tests for any illness and each illness type individually were estimated and are reported in the forest plot in Fig. 2.

The COVID-19 restrictions study period was associated with decreased odds of owners reporting that their dogs had an episode of coughing (0.20, 95% CI: 0.04–0.92) but was not associated with a change in odds of owners reporting that their dogs had an episode of any illness, vomiting, diarrhoea, scratching, licking or chewing, limping or lameness, ear problems, eye problems, skin problems or accident or injury. The COVID-19 restrictions study period was associated with decreased odds of owners reporting that they took their dogs to a veterinarian with an episode of any illness (0.58, 95% CI: 0.45–0.76), scratching (0.46, 95% CI: 0.23–0.96), limping or lameness (0.56, 95% CI: 0.33–0.97), eye problems (0.12, 95% CI: 0.02–0.94) and accident or injury (0.17, 95% CI: 0.03–0.90) but was not associated with a change in odds of owners reporting that they took their dogs to the veterinarian with an episode of vomiting, diarrhoea, coughing, licking or chewing, ear problems or skin problems.

## Discussion

This study established that the COVID-19 restrictions study period was associated with differences in Labrador retrievers’ lifestyle, routine care, insurance status, illness incidence and veterinary attendance for those dogs who were living in England and enrolled in Dogslife. To our knowledge, this is the first time data from a cohort study has been used to investigate associations between COVID-19 restrictions and canine lifestyles, health and veterinary care.

This study estimates that Dogslife owners reported that they exercised their dogs for slightly longer time periods after the COVID-19 restrictions study period. This result initially seems in contrast with surveys of dog owners internationally, where dog owners reported their dogs had fewer walks [40], in Belgrade, where the number of minutes dog owners spent walking their dogs decreased [37] and in the UK, where dogs were walked less frequently and closer to home, but the number of minutes they were exercised for remained the same [16]. However, the exercise reported in Dogslife was transformed from a categorical to a continuous variable prior to analysis and averaged across several different types of exercise. Research from Dogs Trust reported that dog owners played with or trained their dogs more frequently in lockdown, but the number of minutes and frequency of walks decreased and more time was spent walking on a lead, although the effect of breed was not accounted for [41]. Therefore, it could be that Dogslife Labrador retrievers were indeed walked less or similar amounts, but owners compensated with other forms of exercise due to spending more time with their dogs at home.

While to date there has been no peer reviewed research into dogs’ diet during lockdown, there have been several reports in the media of dog owners titbit-feeding more frequently during lockdown. The pet food brand Natural Instinct reported that 44% of the dog owners they surveyed gave their dogs more treats during lockdown [65], over half of owners surveyed by the charity Guide Dogs reported giving their dogs more treats [66] and 34% of dog owners surveyed by insurance group More Than reported giving their pets more treats [67]. These surveys are in contrast with results from this study, which found decreased odds of owners reporting that they fed their dogs titbits SLV during the COVID-19 restrictions study period. An explanation is that a large proportion of Dogslife dogs were working, sporting or guide dogs (Table 1). Owners would not have been able to take their dogs to sporting activities or training classes during lockdown, which may have reduced the number of titbits given as rewards. In support of this theory, one survey of 1833 Italian dog owners reported that 26% gave their dogs treats as a reward during training and sports activities [68] and a qualitative analysis of another survey in the UK revealed that some owners felt that treats should only be given as part of training [69]. However, as Dogslife does not collect information on the reasons behind titbit-feeding or how regularly both pets and working, sporting or guide dogs are taken to training classes, it is impossible to infer whether a reduction in these activities was likely to cause such a large drop in the odds of titbit-feeding by owners.

The odds of owners reporting they had wormed their dog SLV increased during the COVID-19 restrictions study period. Despite the fact that many veterinary practices had limited services, they were still able to dispense wormers for dog owners to collect [53] and owners were still able to use over the counter worming medications. Furthermore, one survey reported that only 69.3% of dog owners in the UK sought advice from their veterinarian about worming [70], so a large proportion of dog owners may have not been affected by changes in veterinary services in terms of their dogs’ worming routines. The higher rates of worming could be explained by improved compliance of owners due to having more time at home with their dogs and being less likely to forget [71]. The results of this study are in contrast with reports of increased ﻿cases of *Angiostrongylus vasorum* lungworm after COVID-19 restrictions began in the UK, which was considered to be a result of a reduction in routine worming treatments [72].

A UK-based survey reported that people who bought puppies during the pandemic were more likely to be first-time pet owners and were more likely to buy from breeders who did not perform health checks on the puppies before sale [7]. Therefore, a rise in inexperienced dog owners who bought from disreputable breeders may have exasperated the problem of poor parasite control measures. New puppy owners may have been reluctant to take them to a veterinarian in the pandemic and may not have received a parasite prevention plan [72]. Furthermore, many veterinary practices were operating on an emergency-only basis [44, 53] and there were reports in the news of veterinary practices not accepting new clients during the pandemic [73], which may have delayed new owners access to veterinary care. The demographics of Dogslife dog owners are considerably different: they buy Kennel Club registered Labrador retrievers and a proportion of Dogslife dogs have another purpose in addition to being pets. Furthermore, the majority of dogs in the COVID-19 restrictions study period were over 1 year of age (Table 1), so most owners had at least some experience of dog ownership and would have probably already been in the routine of parasite prevention.

It is not surprising that there were reduced odds of Dogslife owners reporting that they had vaccinated their dogs SLV during the COVID-19 restrictions study period. There was some confusion about the guidelines from the British Veterinary Association (BVA) about whether veterinary practices should administer vaccinations during the first 2020 lockdown period and while most ceased to administer them, some continued to prevent their emergency services being overwhelmed [74]. The results reported here mirror the reduction in canine vaccination consultations reported by SAVSNET, who reported that the ﻿percentage change in vaccination consultation frequency in dogs during the first social distancing phase of lockdown ﻿(23rd March – 12th April 2020) compared to the same time period in 2019 approached approximately 95% and remained at around 40% by the 4th of July 2020 [63]. Perhaps what is more pertinent is the implications of these results. The WHO have recently warned of the risk of measles outbreaks worldwide due to children missing their routine vaccines during the COVID-19 pandemic [75]. In canine medicine, rises in cases of vaccine preventable diseases such of parvovirus and ﻿leptospirosis after the 2020 lockdown reported by SAVSNET are small yet concerning. Although these diseases currently seem under control, it is not clear whether there will be long-term consequences in immunity for puppies who missed their vaccinations within the optimal time frames, which are recommended to ensure that they are capable of making a primary immune response [76]. Puppies whose vaccinations were postponed also experienced a delay to an important life event: their first veterinary appointment. Positive experiences with veterinarians during puppies’ formative years are vital to prevent later behavioural issues and also provide an opportunity for owners to get advice regarding their socialisation and development [77, 78]. Therefore, these divergences from puppies’ usual appointment timelines may have a long-term impact on their anxiety levels and behaviour.

The odds of owners reporting their dogs being insured were decreased during the COVID-19 restrictions study period, which could be linked to the financial status of owners. The BVA suggested that some owners may deal with the financial pressure of the pandemic in the short-term by cancelling their pet insurance [44]. Furthermore, the price comparison site GoCompare reported a 26% increase in the price of dog insurance in May 2020 in comparison with May 2019, which may have made the decision to cancel or avoid renewing dogs’ insurance easier for some pet owners [79].

The illness incidence of Dogslife dogs did not change during the COVID-19 restrictions study period. However, as this study only included data between the 23rd of March and the 4th of July that were recorded up to a maximum of the 23rd of July in each year, it is possible that the long-term health of dogs was not yet affected. On the other hand, the odds of owners reporting that their dogs experienced a coughing episode during the COVID-19 restrictions study period was greatly reduced to a fifth of previous years. A common cause of coughing in dogs is ﻿canine infectious respiratory disease, also known as “Kennel cough”, which transmits between dogs, has ﻿multiple viral and bacterial pathogens responsible for its causation and is endemic in the population [80, 81]. It is probable that the COVID-19 restrictions led to a reduction in dog socialisation and therefore the transmission of pathogens and that a subsequent reduction in coughing was reported by owners.

The odds of owners reporting that they took their dogs to a veterinarian with an illness episode during the COVID-19 restrictions study period were reduced in comparison with previous years. When specific illnesses were examined, the reduction in odds was still apparent in owners reporting that they took their dogs to a veterinarian with scratching, limping and lameness, eye problems and accident or injury (not adjusted for age). The RCVS reported that in April 2020, 97% of practices it surveyed had limited their services to emergencies and ‘urgent cases’, although how these were defined by different practices were likely to differ and a further 29% had closed either a branch or their main practice [55]. Owners were probably less likely to take their dogs to the veterinarian when they perceived their dogs had a milder problem, such as many cases of scratching or eye problems. They may have also been able to pick up their dogs’ repeat prescriptions for chronic or repetitive illnesses, such as many cases of limping or lameness. Accidents or injuries indicate more immediate problems which may require urgent veterinary treatment. However, this study did not differentiate between mild or more serious types of illness or identify chronic illnesses, so it is impossible to comment whether this was the case.

Alternatively, owners may have had a telephone consultation with their veterinarian about these complaints. In Dogslife, owners are asked “Did you take [dog name] to your vet for the [illness type]?” rather than if the owner received any veterinary advice or treatment. A limitation of this study is that the measurement of how veterinary care was accessed relied on Dogslife owners’ perception of what ‘taking their dog to the vet’ entails. SAVSNET reported an increase in telephone consultations up to an average of about 3% of total consultations in the first and second phases of lockdown ﻿(23rd March – 10th May 2020) [58]. This initially seems like a small percentage and if this estimation is accurate for Dogslife dogs it does not explain the large reduction in veterinary visits reported by owners. However, SAVSNET acknowledge these figures are likely to be an underestimation due to differences in the workflow and data recording of veterinary practices [58]. In support of this, the RCVS survey reported that 100% of 451 practices that answered the question used ﻿remote consulting for existing clients, whilst 45% used it for new clients [55]. Furthermore, HealthforAnimals reported that in a survey of 3258 pet owners in the US, UK, France and Brazil, 27% delayed or avoided contacting their veterinarians and the percentage of owners whose veterinary practices offered digital or remote services rose from 20 to 47% [82]. Similarly, a survey of veterinarians in California reported that practices providing telemedicine services rose from 12% prior to the pandemic to 38% between March the 15th and June the 15th 2020 [83]. It is probable that the results in this study can be explained by a combination of Dogslife owners contacting their veterinarian less often due to concerns related to pandemic, experiencing an increase in telemedicine at their veterinary practices and having difficulties accessing veterinary care.

This study had several limitations which have not yet been discussed. We recommend that readers do not rely solely on the *P*-values reported to infer statistical significance and interpret our findings, but consider the confidence intervals and other results [49]. Questionnaire data such as Dogslife is limited by social desirability bias (the tendency of survey and interview respondents to give answers they feel will be socially acceptable rather than those which reflect the truth) and ‘recall decay’ (the decrease in participants’ ability to accurately recall events as time to reporting increases). Previous Dogslife studies have attempted to account for recall decay [84], but such methods introduce subjective cut-offs and limit the data available, which was not deemed appropriate in the current study. An arbitrary cut-off was used to allow for some delay in illness reporting by owners and it is unlikely to have fully captured the true number of illnesses reported that were experienced in the study period. However, this was used for both the COVID-19 restrictions study period and the data in the same date range in previous years. A limitation of Dogslife postcode data is that it may not be accurate, because owners are only reminded via annual newsletters to update a change of address, or by the Dogslife secretary when it is apparent that an address is out of date. Therefore, it is possible that a small proportion of owners’ postcodes may not have been resident in England for the dates that questionnaires were uploaded to the Dogslife website. However, as widespread lockdown restrictions occurred over the entirety of the UK, any inaccuracies are unlikely to have affected the results.

Furthermore, attrition (the loss of participants during the course of the study) may have affected the results of this study. It is typical for some Dogslife owners to not report for a long period between one questionnaire and the next one. This makes it difficult to determine the true attrition rates for Dogslife and all owners were considered as ‘available to report’, when this was unlikely. The illnesses included in this study were reported by owners and had not received veterinary diagnoses, so they probably had a wide variety of aetiologies. Dogslife is a study of Labrador retrievers and the results reported here may not be generalisable to other dog breeds. Additionally, as has partly been discussed previously, the demography, dog owning experience and behaviour of Dogslife owners may differ from the general population of UK dog owners, especially during the COVID-19 restrictions study period with the increase of new dog owners. Finally, the cleaning, categorising and coding of the data included is subjective and relies on the researcher’s expertise and opinion.

## Conclusion

This study demonstrates that COVID-19 restrictions were associated with differences in Labrador retrievers’ lifestyle, routine care, insurance status, illness incidence and veterinary attendance who were living in England. Dogslife provided a unique opportunity to study prospective questionnaire data from owners already enrolled on a longitudinal cohort study, which is likely to minimise biases associated with recalling events prior to the pandemic. Furthermore, Dogslife includes a wider population of dogs than can be studied in primary care data and provides insights into owners’ decision making about their dogs’ healthcare. The implications of the changes to dogs’ lives have not been fully realised, but future research should aim to elucidate how negative impacts of the COVID-19 pandemic and associated restrictions on dogs can be minimised. This will inform owners and veterinarians of the best practices for keeping dogs healthy in future pandemics, which are becoming increasingly more frequent and severe.

## Methods

### Collection and selection of Dogslife data

Dogslife is a longitudinal online study of the health of pedigree UK Kennel Club registered Labrador retrievers. Recruitment to Dogslife began in July 2010 and continues at the time of writing. Extensive details of the study design have been published previously [64, 85]. Dog owners register to Dogslife when their dogs are aged under one year and supply demographic and geographic information. They are then asked to complete online questionnaires about their dogs’ morphology, lifestyle and illness incidences every month when their dogs are under the age one and every three months when their dogs are over the age of one. No attempt was made to identify if owners were more or less likely to report based on their current level of participation in Dogslife and all owners were thus equally considered as ‘available to report’ at any time in the study.

Data for this study were collected from dogs living in England during the first 10 years of Dogslife, via routine online reporting. Dogs in other areas of the UK were not included into the study due to national differences in COVID-19 restrictions and because the majority of participants (80.02%) in the Dogslife study live in England. Postcode data supplied by owners were used to select participants. These were classified into areas of the UK and verified using data publicly available from the Office for National Statistics [86]. Data from the Office for National Statistics is licensed under the Open Government Licence V. 3.0.

The 23rd of March 2020 was selected as the study onset date because it was when the first lockdown restrictions were introduced in England. The 4th of July 2020 was selected as the study conclusion date because lockdown restrictions were eased considerably, resulting in the opening of public houses and restaurants and the beginning of localised lockdown restrictions in England [3]. For clarity, this time period is referred to as the ‘COVID-19 restrictions study period’. Data between the 23rd of March and the 4th of July in previous years from 2011 to 2019 were used as a comparison to data from the COVID-19 restrictions study period.

### Data cleaning and analysis

Dogslife data was cleaned and assessed for quality prior to analysis and duplicates were removed while maximising the information they contained by coalescing where information was missing between the otherwise duplicated entries. Quantifying missing data within the Dogslife dataset is complex due to the challenges of handling very large datasets. In many instances, data are not ‘missing’ in the sense that they are unobserved but can be absent as relevant events, removed during data cleaning, or auto-filled as missing by default. The authors can be contacted directly to obtain this information if needed. Specific data cleaning of variables is discussed in more detail within the relevant methods sections. Additional research showed that the frequency with which owners’ reported to Dogslife did not to differ in the COVID-19 restrictions study period in comparison with data in the same date range in previous years, so the potential of a confounding effect due to differences in reporting frequency was ruled out prior to other analyses. An additional file 2 gives details of this analysis.

All data analysis was carried out using R statistical software (R version 4.1.1). An example of the code, including the specific packages and functions used for this study are available in a public repository [87]. All data are reported to four significant figures. An additional file 3 gives a checklist for the STrengthening the Reporting of OBservational studies in Epidemiology (STROBE) [88] reporting guidelines, which were adhered to in this study.

### Modelling the association of COVID-19 restrictions with the lifestyle and routine care of Dogslife dogs

Dogslife questionnaire data that summarise the lifestyle and routine care of the cohort were selected based on the perceived plausibility that they might have been affected by the lockdown restrictions. Data were transformed where necessary for modelling purposes into variables of interest (See Table 2). Questions about dogs’ exercise quantities totalled 12 questions and seven answer categories, and modelling each of these would have increased the chance of a false discovery due to multiple comparisons [89]. Additionally, multinomial regression assumes the normality of independent variables and the linearity of relationships, which were violated using our data [90]. Therefore it was considered appropriate to transform exercise quantity from several categorical variables to continuous variables so that they could be easily combined into a single variable to be used as an outcome in continuous regression models. A randomised value based on the normal distribution was assigned between the bottom and top limit of each daily time quantity category. For the category ‘over 2 hours’, a top limit of four hours of exercise per day was assumed, so that a random value between 2 hours and 4 hours was assigned for this particular category. Weekday and weekend exercise quantities were combined by multiplying weekday quantities by 5/7 and weekend quantities by 2/7 and summing these values to produce a weighted average. The sum of all averaged exercise variables was calculated to produce a total ‘exercise quantity’ in minutes of per exercise week. Dried food was chosen as the indicator of food quantity because Dogslife owners report feeding dried food to their dogs more frequently than other types of food and because the consistency and weight of dried food is less variable than wet food. Dried food quantity was cleaned using an adapted version of the published and validated ‘NLME-A’ method [91]. In brief, non-linear mixed models were combined with an algorithm that used cut-off values based on recommendations for dried food intake for male and female Labrador retriever puppies from the most popular dried food brand in Dogslife, which was Royal Canin [92].

**Table 2:** Variables of interest derived from Dogslife questionnaires relating to lifestyle, routine care and insurance status

Name of variable	Relevant Dogslife questionnaire question(s) and answer(s) reported by owners	Description of variable
Exercise quantity	Q: On average, in the last week for how long does [dog name] do the following exercise(s) EACH day (Weekday/Weekend): Walking on the lead? Running on the lead? Walking/running off the lead? Exercise involving fetching, chasing or retrieving? Obedience training? Other playing activity (including dogs playing together)? A: [None, 1–5 min, 5–15 min, 15–30 min, 30–60 min, 1–2 hrs, Over 2 hrs]	Multiple categorical Dogslife questions transformed to a single continuous variable to estimate dogs’ minutes of exercise per week
Dried food quantity	Q: How much dried food do you feed [dog name] in total each day? A: [Free text with unit box]	Continuous variable converted into grams of dried food fed to dogs per day reported by owners
Insurance status	Q: Is [dog name] insured? A: [Yes/No]	Binomial yes/no variable
Titbits status	Q: Does [dog name] also receive ‘titbits’? For example anything else [dog name] eats such as food off your plate, training treats, chews etc. A: [Yes/No]	Binomial yes/no variable
Sleep-person status	Q: Where does [dog name] sleep at night? A: [Alone in a room in a house, In a room shared with a person, In a room shared with a pet, In a room shared with a pet and a person, Outside, Other]	Transformed into a single binomial yes/no variable to categorise whether dogs sleep with a person at night
Bathed SLV^a^	Q: Has [dog name] been bathed since you last visited the site? A: [Yes/No]	Binomial yes/no variable
Anti-parasitic SLV^a^	Q: Have you used any products to prevent or treat fleas or ticks since you last visited the site? A: [Yes/No]	Binomial yes/no variable
Wormed SLV^a^	Q: Has [dog name] been wormed since you last visited the site? A: [Yes/No]	Binomial yes/no variable
Vaccinated SLV^a^	Q: Has [dog name] been vaccinated since you last visited the site? A: [Yes/No]	Binomial yes/no variable

^a^Since the owner’s last visit to the Dogslife website (SLV)

Various regression models were fitted to continuous variables of interest (exercise quantity and dried food quantity) and binomial variables of interest (insurance status, titbits status, sleep-person status, bathed SLV, anti-parasitic SLV, wormed SLV and vaccinated SLV). The final models chosen were GAMMs based on comparison of the models using various diagnostic techniques. Models for continuous variables of interest had Gaussian distributions and models for binomial variables of interest had binomial distributions with logit links. An independent variable was added to the models to compare the COVID-19 restrictions study period to data in the same date range in previous years. A smooth term for dog age was fitted to control for non-linear age effects, the sex of the dogs was included to control for sex effects and a random effects (RE) term was added for dog identification (ID) to control for individual effects. Dog age was calculated to the date that owners entered questionnaire data into the Dogslife website.

### Analysing the association of COVID-19 restrictions with illness incidence and associated veterinary attendance in Dogslife

Dogslife questionnaire data related to dogs’ illness incidences, the start dates of illness and dogs’ veterinary attendance with illness incidences were selected. (See Table 3). When illness start dates were suspected to be erroneous (e.g. when the dates were before dogs’ dates of birth), they were corrected (e.g. when dates were suspected to be a unit error away from the correct dates) or removed.

**Table 3:** Information derived from Dogslife questionnaires relating to dogs’ illness and veterinary attendance status

Relevant Dogslife questionnaire question(s)	Information derived
Q: Has [dog name] had any of the following problems: Vomiting? Diarrhoea? Coughing? Scratching themselves? Licking or chewing themselves? Limping or lameness? Did [dog name] have any other illnesses or problems? A: [Yes/No]	Illness incidences
Q. Approximately when did the vomiting start? A: [Calendar box]	Start date for illnesses
Q. Did you take [dog name] to your vet for the [illness type]? A: [Yes/No]	Veterinary attendance with an illness incidences

The incidences of illness and veterinary attendance with an illness were first investigated as a total count of questionnaires where owners reported any illness or any veterinary attendance with an illness, respectively and illnesses were then further classified into ten categories. The first six illness categories were routinely reported by owners in each Dogslife questionnaire: vomiting, diarrhoea, coughing, scratching, licking or chewing and limping or lameness. The final four illness categories were derived from the ‘other illness’ category of Dogslife questionnaires. The primary researcher (CSCW) manually coded other illnesses and the four most frequently reported were selected for inclusion into the study: eye problems, ear problems, skin problems and accident or injury.

From the beginning of Dogslife in July 2010 to the 1st of August 2020, there was a median reporting time lag of 19 days (Interquartile range: 35) between the illness start dates given by owners and the date that owners entered questionnaire data into the Dogslife database. To allow for some owner delay in illness reporting, 19 days were added onto the study conclusion date (the 4th of July became the 23rd of July in each year) and questionnaires were included up to this date. Where the illness start dates given by owners were available (85.69%), they were used to exclude illnesses that were before the 23rd of March and after the 4th of July from 2011 to 2020.

Chi-squared tests were performed to compare the incidences of illness and associated veterinary attendance in the COVID-19 restrictions study period to data in the same date range in previous years. To account for the aging population of the Dogslife cohort, these tests were performed with age stratification using the Mantel-Haenszel (MH) adjustment into the following four age groups: under 1 year, 1 to 3.49 years, 3.5 to 6.99 years and 7 years or over. The age groups were arbitrarily defined to separate dogs under one year due to differences in Dogslife reporting, to divide the remaining data within the confines of Dogslife (where the oldest dog was less than 11 years old) and to approximately capture the four periods of Labrador retriever aging as reported by Wang and colleagues [93]. The MH adjustment was not performed for veterinary attendance with coughing and accident or injury because expected frequencies of the stratified groups contained values < 5 and chi-square tests are inaccurate at small numbers of expected frequencies [94, 95]. Dog age was calculated to the illness start dates given by owners where they were available (85.69%) and otherwise to the date that owners entered questionnaire data into the Dogslife database.

## Supplementary Information

**Additional file 1.**

**Additional file 2.**

**Additional file 3.**

## Data Availability

The datasets generated and/or analysed during the current study are not publicly available because individual privacy may be compromised but are available from the corresponding author on reasonable request.
