# Supplementary material for: The impact of the COVID-19 pandemic on a cohort of Labrador retrievers in England
Source: BMC Vet Res. 2022 Jun 24;18:246. doi: 10.1186/s12917-022-03319-z (PMC9233325; doi:10.1186/s12917-022-03319-z)
Supplement: Supplementary file 1 — Additional file 1. [file 12917_2022_3319_MOESM1_ESM.pdf]

File name: Additional file 1

File format: PDF

Title: Model selection, diagnostics, fit and full output.

Description: A file containing supplementary tables and figures showing the model selection process, model diagnostics and fit and the full output of models' estimates fitted to variables of interest relating to Dogslife dogs' lifestyle, routine care and insurance status

Table S1: Model fit indicators for models fitted to variables of interest relating to lifestyle, routine care and insurance status

Variable of interest and model distribution	AIC/RMSE/ AUC with 95% CI	Linear mixed models	General additive mixed models with a smooth term for dog age
Exercise quantity (Gaussian)	AIC	162514.3	161161.0
	RMSE	62.11	58.58
Dried food quantity (Gaussian)	AIC	154150.5	153519.7
	RMSE	66.54	64.85
Insurance status (Binomial)	AIC	5372.90	5374.91
	AUC<br>95% CI:	99.53%<br>99.47%-99.6%	99.53%<br>99.47%-99.6%
Titbits status (Binomial)	AIC	10245.13	10095.53
	AUC<br>95% CI:	97.10%<br>96.86%-97.35%	97.34%<br>97.09%-97.58%
Sleep-person status (Binomial)	AIC	6595.422	6337.928
	AUC<br>95% CI:	99.42%<br>99.34%-99.50%	99.50%<br>99.43%-99.58%
Bathed SLV* (Binomial)	AIC	16316.16	16089.51
	AUC<br>95% CI:	91.47%<br>91.01%-91.93%	92.11%<br>91.67%-92.55%
Anti-parasitic SLV* (Binomial)	AIC	16909.3	16755.66
	AUC<br>95% CI:	89.56%<br>89.03%-90.09%	89.56%<br>89.03%-90.09%
Wormed SLV* (Binomial)	AIC	17992.42	17841.94
	AUC<br>95% CI:	84.96%<br>84.31%-85.6%	84.96%<br>84.31%-85.6%
Vaccinated SLV* (Binomial)	AIC	15503.27	13210.2
	AUC<br>95% CI:	88.59%<br>88.02%-89.15%	88.65%<br>88.06%-89.24%

The Akaike information criterion (AIC) and the root-mean-square error (RMSE) of gaussian models and the AIC and percentage area under the curve (AUC) with 95% confidence interval (CI) of binomial models (with logit links) fitted to variables of interest, for comparing the COVID-19 restrictions study period (March 23rd to July 4th 2020) to data in the same date range in previous years (March 23rd to July 4th, 2011 to 2019) and adjusted for age, sex and individual effects. *Since the owner's last visit to the Dogslife website (SLV)

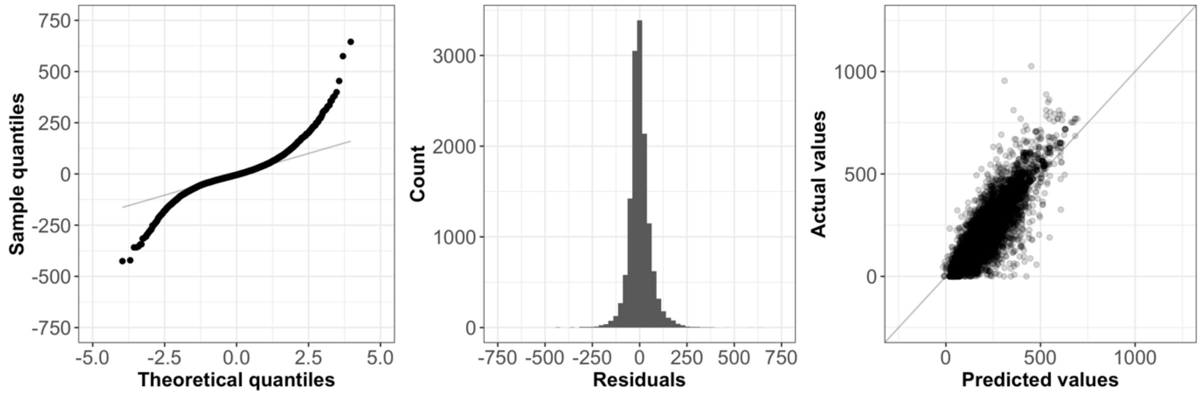

Figure S1: Diagnostic plots for a general additive model fitted to Dogslife dogs' exercise quantity. The quantile-quantile plot, residual histogram and predicted VS actual plots for a general additive model fitted to the exercise quantity (minutes per week) of Dogslife dogs, for comparing the COVID-19 restrictions study period (March 23rd to July 4th 2020) to data in the same date range in previous years (March 23rd to July 4th, 2011 to 2019) and adjusted for age, sex and individual effects

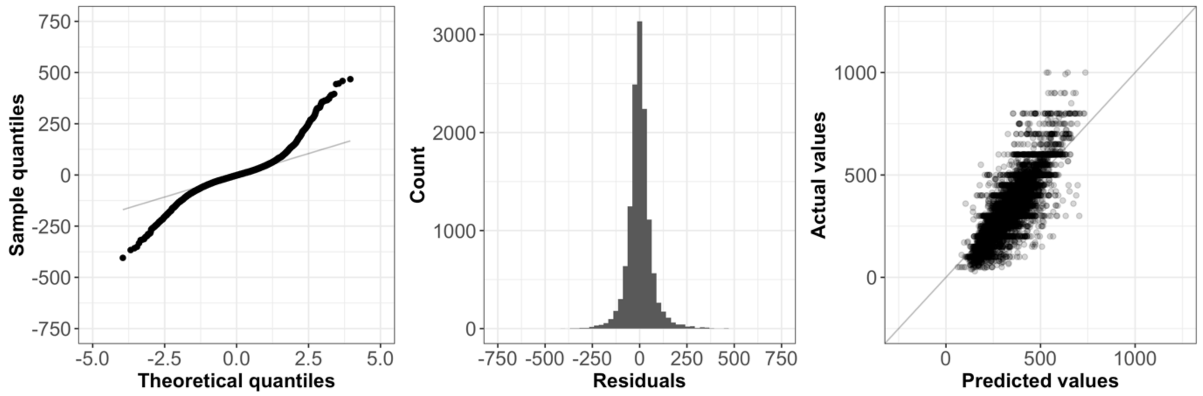

Figure S2: Diagnostic plots for a general additive model fitted to Dogslife dogs' dried food quantity. The quantile-quantile plot, residual histogram and predicted VS actual plots for a general additive model fitted to the dried food quantity (grams per day) of Dogslife dogs, for comparing the COVID-19 restrictions study period (March 23rd to July 4th 2020) to data in the same date range in previous years (March 23rd to July 4th, 2011 to 2019) and adjusted for age, sex and individual effects

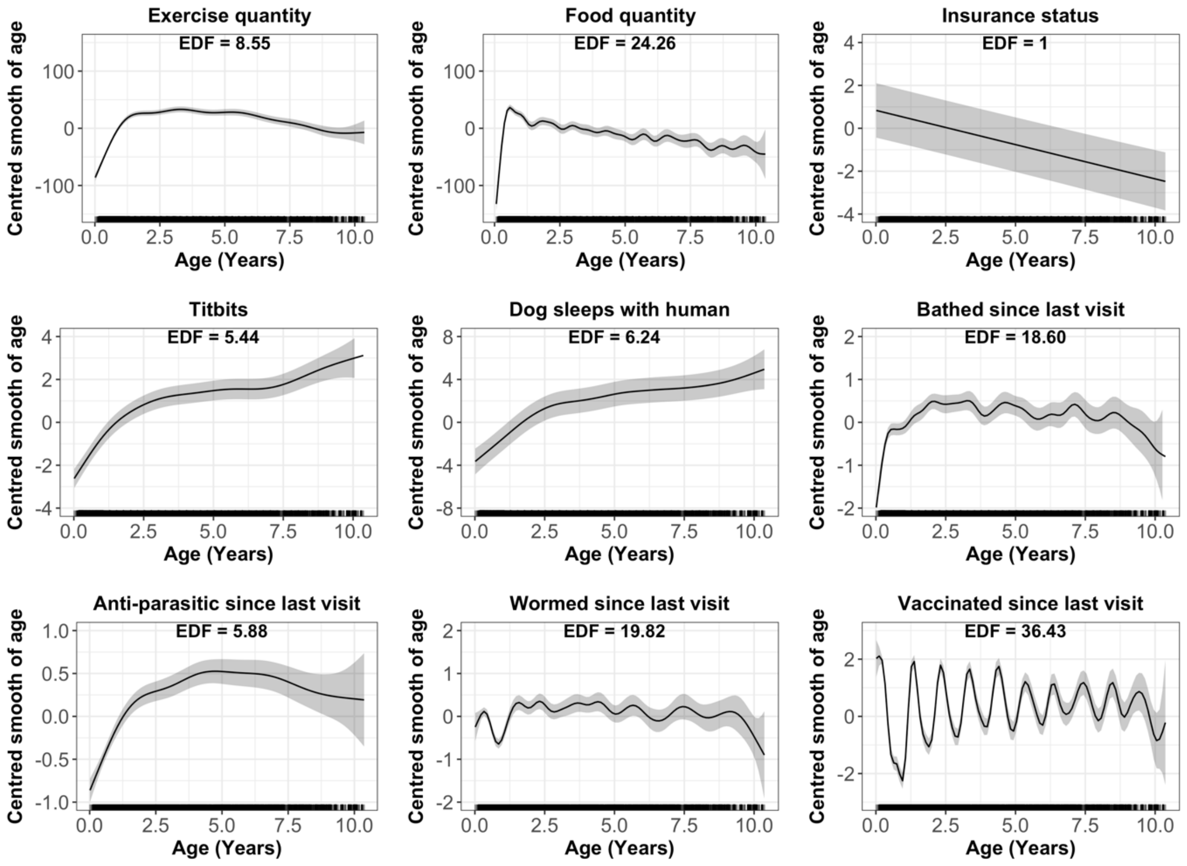

Figure S3: General additive model curves fitted to variables of interest. The fitted smoothed general additive models' curves for dog age and their estimated degrees of freedom (EDF) fitted to variables of interest, for comparing the COVID-19 restrictions study period (March 23rd to July 4th 2020) to data in the same date range in previous years (March 23rd to July 4th, 2011 to 2019) and adjusted for age, sex and individual effects

Table S5: Parameter estimates for general additive models fitted to variables of interest

Variable of interest and model distribuition	Predictors	BC/OR	95% CI	P-Value
Exercise quantity (Gaussian)	COVID-19 restrictions study period	6.89	1.65 – 12.13	0.010
	Sex of dog is male	4.89	-1.61 – 11.38	0.140
	Smoothed age of dogs	- - -	- - -	<0.001
Dried food quantity (Gaussian)	COVID-19 restrictions study period	-0.88	-6.69 – 4.93	0.766
	Sex of dog is male	39.71	33.07 – 46.35	<0.001
	Smoothed age of dogs	- - -	- - -	<0.001
Insurance status (Binomial)	COVID-19 restrictions study period	0.59	0.39 – 0.90	0.015
	Sex of dog is male	1.59	0.81 – 3.12	0.181
	Smoothed age of dogs	- - -	- - -	<0.001
Titbits status (Binomial)	COVID-19 restrictions study period	0.36	0.24 – 0.52	<0.001
	Sex of dog is male	1.02	0.69 – 1.53	0.908
	Smoothed age of dogs	- - -	- - -	<0.001
Sleep-person status (Binomial)	COVID-19 restrictions study period	1.07	0.68 – 1.68	0.771
	Sex of dog is male	2.39	1.18 – 4.86	0.016
	Smoothed age of dogs	- - -	- - -	<0.001
Bathed SLV* (Binomial)	COVID-19 restrictions study period	0.99	0.82 – 1.20	0.958
	Sex of dog is male	1.31	1.12 – 1.53	0.001
	Smoothed age of dogs	- - -	- - -	0.017
Anti-parasitic SLV* (Binomial)	COVID-19 restrictions study period	1.00	0.84 – 1.20	0.990
	Sex of dog is male	1.00	0.88 – 1.14	0.964
	Smoothed age of dogs	- - -	- - -	0.003
Wormed SLV* (Binomial)	COVID-19 restrictions study period	1.20	1.02 – 1.40	0.028
	Sex of dog is male	0.98	0.88 – 1.09	0.706
	Smoothed age of dogs	- - -	- - -	0.841
Vaccinated SLV* (Binomial)	COVID-19 restrictions study period	0.70	0.58 – 0.83	<0.001
	Sex of dog is male	1.13	1.00 – 1.27	0.051
	Smoothed age of dogs	- - -	- - -	0.270

General additive model estimates for the beta coefficient (BC) (for gaussian models) or odds ratios (OR) (for binomial models), 95% confidence interval (CI) and P-values, fitted to variables of interest, for comparing the COVID-19 restrictions study period (March 23rd to July 4th 2020) to data in the same date range in previous years (March 23rd to July 4th, 2011 to 2019) and adjusted for age, sex and individual effects
