# Supplementary material for: The impact of the COVID-19 pandemic on a cohort of Labrador retrievers in England
Source: BMC Vet Res. 2022 Jun 24;18:246. doi: 10.1186/s12917-022-03319-z (PMC9233325; doi:10.1186/s12917-022-03319-z)
Supplement: Supplementary file 2 — Additional file 2. [file 12917_2022_3319_MOESM2_ESM.pdf]

File name: Additional file 2

File format: PDF

Title: Investigating the association of COVID-19 restrictions with Dogslife owner reporting frequency.

Description: To rule out a potential confounding effect in the main research questions, the reporting frequency of Dogslife owners in the COVID-19 restrictions study period was compared to data in the same date range in previous years. This file contains details of this supplementary analysis

Aim

To rule out a potential confounding effect in the main research questions, the reporting frequency of Dogslife owners in the COVID-19 restrictions study period was compared to data in the same date range in previous years.

Methods

A total of 16115 Dogslife questionnaires from 4110 dogs, entered into the Dogslife website by owners between March 23rd and July 23rd, 2011 to 2020 were included in analysis. The number of Dogslife questionnaires were calculated between the dates March 23rd to July 23rd for each year from 2011 to 2020 to produce 10 values. To account for differences in reporting due to the total number of dog registrations to Dogslife at different dog ages, the counts of dogs registered on the mid-date of these 10 time periods (24th May) were calculated and categorised into dogs under one year of age or equal to and above one year of age on this date (Table S3).

Table S3: Data included in analysis

Year	Number of questionnaires reported between<br><br>March 23rd and July 23rd	Annual cumulative registrations of dogs aged under one year	Annual cumulative registrations of dogs aged equal to or over one year
2011	1330	625	209
2012	1410	515	1126
2013	1698	547	1927
2014	1718	444	2730
2015	1892	433	3421
2016	1726	400	4094
2017	1673	391	4718
2018	1642	281	5346
2019	1491	63	5650
2020	1535	80	5775

Various Poisson distribution (with log link) generalised linear models (GLMs) and generalised additive models (GAMs) were fitted to the number of questionnaires reported between March 23rd and July 23rd in each year from 2011 to 2020. An independent variable was added to the models to compare

the COVID-19 restrictions study period to data in the same date range in previous years. Smooth terms for cumulative registration counts for dogs under and over one year of age were fitted to allow for non-linear relationships between registration counts at these ages and the number of questionnaires.

Results

The final model chosen was a GAM based on comparison of the models using various diagnostic techniques including minimising the value of the Akaike information criterion (AIC) and the root-mean-square error (RMSE) (Table S4) and by visualising diagnostic plots. The diagnostic plots of the final model are shown in Figure S4. The fitted GAM curves and estimated degrees of freedom (EDF) are shown in Figure S5. The full model estimates are shown in Table S5.

The incidence rate ratio for the COVID-19 restrictions study period was not associated with a difference in the number of questionnaires reported to Dogslife between the dates March 23rd to July 23rd in each year from 2011 to 2020,

Table S4: Model fit indicators for Poisson models fitted to the number of Dogslife questionnaires

AIC/ RMSE	Poisson general linear model	Poisson general linear model with quadratic terms for dog registrations under and over 1 year old	Poisson general additive model with smooth terms for dog registrations under and over 1 year old
AIC	177.09	117.43	111.73
RMSE	112.71	47.71	32.92

The Akaike information criterion (AIC) and the root-mean-square error (RMSE) were calculated for three different Poisson models (with log links) fitted to the number of Dogslife questionnaires reported between March 23rd and July 23rd in each year from 2011 to 2020 and adjusted for dog registrations under and over 1 year old

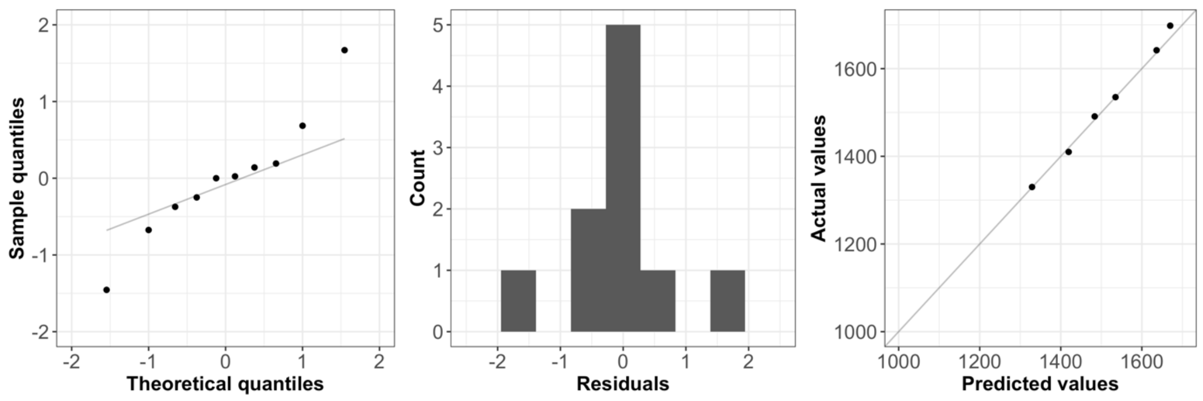

Figure S4: Diagnostic plots for a Poisson general additive model fitted to the number of Dogslife questionnaires. The quantile-quantile plot, residual histogram and predicted VS actual plots for a Poisson general additive model, fitted to the number of Dogslife questionnaires reported, for comparing the COVID-19 restrictions study period (March 23rd to July 4th 2020) to data in the same date range in previous years (March 23rd to July 4th, 2011 to 2019) and adjusted with smooth terms for dog registrations under and over 1 year old

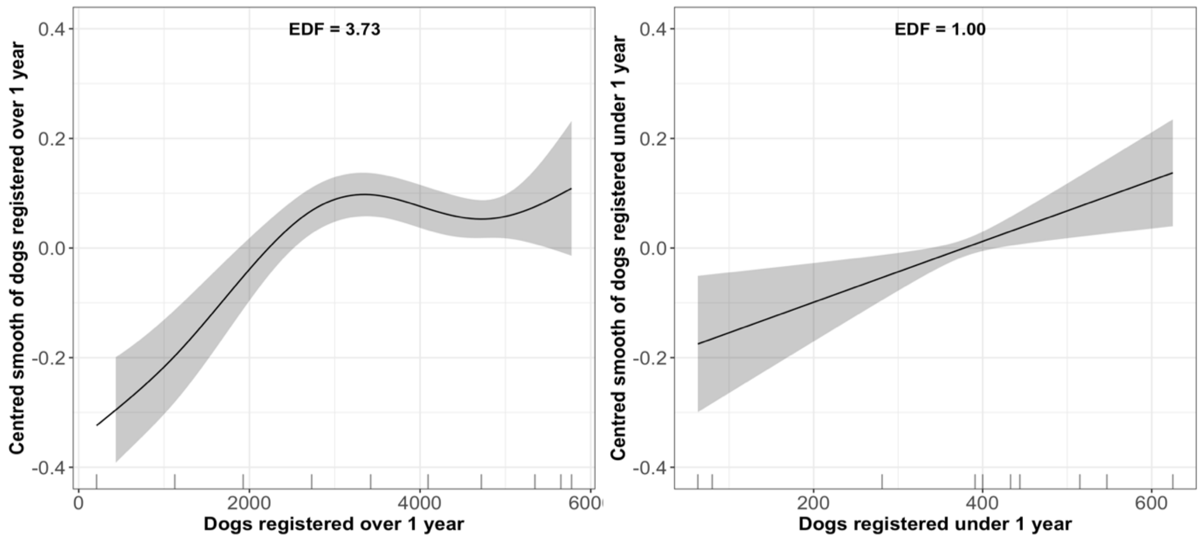

Figure S5: Poisson general additive model curves fitted to the number of Dogslife questionnaires. The fitted smoothed general additive model curves for dogs registered under and over 1 year old and their estimated degrees of freedom (EDF) in general additive models fitted to the number of Dogslife questionnaires reported, for comparing the COVID-19 restrictions study period (March 23rd to July 4th 2020) to data in the same date range in previous years (March 23rd to July 4th, 2011 to 2019) and adjusted with smooth terms for dog registrations under and over 1 year old

Table S5: Parameter estimates for a Poisson general additive model fitted to the number of Dogslife questionnaires

Predictors	IRR	95% CI	P-Value
COVID-19 restrictions study period	1.01	0.95 – 1.09	0.691
Smoothed dogs registered equal to or over 1 year old	- - -	- - -	<0.001
Smoothed dogs registered under 1 year old	- - -	- - -	0.005

Poisson general additive model estimates for the incidence rate ratio (IRR), 95% confidence interval (CI) and P-values, fitted to the number of Dogslife questionnaires reported, for comparing the COVID-19 restrictions study period (March 23rd to July 4th 2020) to data in the same date range in previous years (March 23rd to July 4th, 2011 to 2019) and adjusted with smooth terms for dog registrations under and over 1 year old
